# A Simple Algorithm for Higher-Order Delaunay Mosaics and Alpha Shapes

**DOI:** 10.1007/s00453-022-01027-6

**Published:** 2022-08-28

**Authors:** Herbert Edelsbrunner, Georg Osang

**Affiliations:** IST Austria (Institute of Science and Technology Austria), Am Campus 1, 3400 Klosterneuburg, Austria

**Keywords:** Delaunay mosaics, Voronoi tessellations, Algorithms, Software, Computational experiments

## Abstract

We present a simple algorithm for computing higher-order Delaunay mosaics that works in Euclidean spaces of any finite dimensions. The algorithm selects the vertices of the order-*k* mosaic from incrementally constructed lower-order mosaics and uses an algorithm for weighted first-order Delaunay mosaics as a black-box to construct the order-*k* mosaic from its vertices. Beyond this black-box, the algorithm uses only combinatorial operations, thus facilitating easy implementation. We extend this algorithm to compute higher-order $\alpha$-shapes and provide open-source implementations. We present experimental results for properties of higher-order Delaunay mosaics of random point sets.

## Introduction

Order-*k* Voronoi tessellations are a generalization of ordinary Voronoi tessellations. Instead of each domain corresponding to a single point in the input, $A \subseteq {\mathbb {R}}^d$, each order-*k* domain corresponds to a subset, $Q \subseteq A$, of size *k*, and consists of the set of points $x \in {\mathbb {R}}^d$ for whom the points in *Q* are the closest *k* points within *A*. Its dual is the order-*k* Delaunay mosaic. We will formally define both in Sect. 2. Order-*k* Voronoi tessellations were introduced by [22] as a data structure for fast *k* closest point queries, namely in time $O(k + \log n)$ with $n = \#{A}$. A less direct application is the computation of the distance-to-measure introduced in [6] and related to *k* closest point search in [14]. Furthermore, certain subcomplexes of the order-*k* Delaunay mosaic realize the order-*k* $\alpha$-shapes introduced in [15]. Order-*k* $\alpha$-shapes are a generalization of $\alpha$-shapes [10] used to approximate the shape of a point set. Unlike ordinary $\alpha$-shapes and depending on the parameter *k*, they exhibit robustness to noisy data points.

In the plane, the number of domains in the order-*k* Voronoi tessellation or, equivalently, the number of vertices in the order-*k* Delaunay mosaic is $\Theta (k(n - k))$; see [16, 22]. For dimensions $d \ge 3$, this number can vary significantly depending on the way the input points are distributed. The upper bound of $O(k^{\lceil \frac{d+1}{2} \rceil } n^{\lfloor \frac{d+1}{2} \rfloor })$ on the total size of the first *k* higher-order Delaunay mosaics [7] is tight, while the lower bound of $\Omega (k^d n)$ [18] is only conjectured. For individual order-*k* Delaunay mosaics, the complexity is poorly understood. The problem is closely related to the $(d+1)$-dimensional *k*-set problem. Specifically, the points in $A \subseteq {\mathbb {R}}^d$ can be mapped to equally many points in ${\mathbb {R}}^{d+1}$ such that the order-*k* domains in ${\mathbb {R}}^{d}$ correspond to *k*-sets in ${\mathbb {R}}^{d+1}$, see e.g. [7].

The first algorithm to compute order-*k* Voronoi tessellations and Delaunay mosaics in the plane was described by Lee [16]. The algorithm computes the Voronoi tessellations one by one, in increasing order and in time $O(k^2 n \log n)$. Mulmuley [18] extended this algorithm beyond two dimensions, computing the first *k* levels in a special $(d+1)$-dimensional hyperplane arrangement, which implicitly yield the order-*k* Voronoi tessellations and Delaunay mosaics in time $O(s \log n + k^d n^2)$, in which *s* denotes the output size. Mulmuley [19] later described another algorithm, which instead adds hyperplanes one by one, and runs in time $O(k^{\lceil \frac{d+1}{2} \rceil } n^{\lfloor \frac{d+1}{2} \rfloor })$ for $d \ge 3$, which equals the worst-case output size. For $d=2$, the expected runtime is $O(k^2 n \log \frac{n}{k})$. Another incremental algorithm with similar complexity for $d \ge 3$ has been described by Agarwal et al. [1].

In this paper, we describe a new algorithm for computing order-*k* Delaunay mosaics in Euclidean space of any finite dimension that stands out in its simplicity. It employs an algorithm for weighted first-order Delaunay mosaics, and otherwise uses only combinatorial operations. It thus benefits from highly optimized existing implementations and, if desired, can build upon their use of exact arithmetic. Its complexity depends on the complexity of the algorithm used for weighted Delaunay mosaics. Assuming it is linear in its output size, then our algorithm computes the order-*k* from the order-$(k-1)$ Delaunay mosaic in time that is linear in the size of the two mosaics, and overall runs in time linear in the total size of the order-*k* Delaunay mosaics from order 1 to *k*. We implement this algorithm and run it on various point sets, shedding light on the size and other properties of order-*k* Delaunay mosaics. In particular, we compare the total size of the first *k* Delaunay mosaics of random point sets with the (tight) worst-case upper bound, and we study the size of individual order-*k* Delaunay mosaics, for which no tight bounds are known in general. As far as we are aware, no such experimental investigations have been performed in the past, possibly due to the lack of a practical algorithm. We extend our algorithm to compute the radius function on an order-*k* Delaunay mosaic, which gives us the subcomplexes realizing order-*k* $\alpha$-shapes. Open-source implementations of our algorithm are available [20, 21].

Our algorithm makes use of the *rhomboid tiling* [11], which we will introduce in Sect. 2 alongside other necessary definitions. We will explore the combinatorial properties of this tiling and, by proxy, the properties of order-*k* Delaunay mosaics in Sect. 3. Using these results, we explain our algorithm in Sect. 4. We present experimental results obtained with two implementations of this algorithm in Sect. 5. Section 6 introduces a radius function on the order-*k* Delaunay mosaics and a way to compute it to yield order-*k* $\alpha$-shapes. We close with a discussion of possible extensions and optimizations in Sect. 7.

## Definitions

**Fig. 1:**
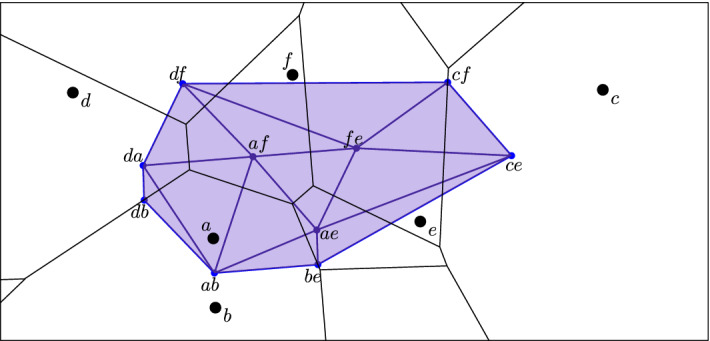
Superposition of the order-2 Voronoi tessellation (in black) and the order-2 Delaunay mosaic (in blue) of a set of six points in the plane. Each domain of the tessellation corresponds to two of these six points, and the corresponding vertex of the mosaic is the average of these two points (Color figure online)

Given a locally finite set, $A \subseteq {\mathbb {R}}^d$, the *(Voronoi) domain* of $Q \subseteq A$ is $\mathrm{dom}{({Q})} = \{x \in {\mathbb {R}}^d \mid \Vert {x}-{q}\Vert \le \Vert {x}-{a}\Vert , \forall q \in Q, \forall a \in A {\setminus } Q \}$. Its *order* is $\#{Q}$. For each positive integer *k*, the *order-k Voronoi tessellation* is $\mathrm{Vor}_{k}{({A})} = \{ \mathrm{dom}{({Q})} \mid Q \subseteq A, \#{Q} = k \}$. The *order-k Delaunay mosaic* is the cell complex dual to $\mathrm{Vor}_{k}{({A})}$, denoted $\mathrm{Del}_{k}{({A})}$. To realize the mosaic geometrically, we usually use the average of the points in *Q* as the location of the corresponding vertex in ${\mathbb {R}}^d$. In a few instances we use the sum rather than the average, for convenience. Figure 1 shows an example for $k=2$. As we will see shortly, in $d \ge 3$ dimensions, the order-*k* Delaunay mosaic is not necessarily simplicial even if the points are in general position.

Assuming *A* is in general position, [11] established the existence of a tiling in ${\mathbb {R}}^{d+1}$ whose horizontal integer slices are the Delaunay mosaics. We recall the definition of the tiling and its most important properties. Let $A \subseteq {\mathbb {R}}^d$ be locally finite and in general position. We construct a rhomboid, $\varrho$, for each partition $A = {{A}}_{ in}\sqcup {{A}}_{ on}\sqcup {{A}}_{ out}$ for which there exists a sphere *S* in ${\mathbb {R}}^d$ such that all points in ${{A}}_{ in}$ lie inside *S*, all points in ${{A}}_{ on}$ lie on *S*, and all points in ${{A}}_{ out}$ lie outside *S*. Whenever convenient, we write ${{A}}_{ in}(\varrho ) = {{A}}_{ in}$, ${{A}}_{ on}(\varrho ) = {{A}}_{ on}$, and ${{A}}_{ out}(\varrho ) = {{A}}_{ out}$ to indicate the correspondence. Due to general position of *A*, we have $\#{{{A}}_{ on}} \le d+1$. A *combinatorial vertex* of $\varrho$ is a collection of points that contains ${{A}}_{ in}$ and is contained in ${{A}}_{ in}\cup {{A}}_{ on}$, and we write

1 $$\begin{aligned} {V}{({\varrho })}&= \{ {{A}}_{ in}\subseteq Q \subseteq {{A}}_{ in}\cup {{A}}_{ on}\} \end{aligned}$$

for the collection of combinatorial vertices of $\varrho$. Setting $y_a = (a, -1) \in {\mathbb {R}}^{d+1}$, for every $a \in A$, we draw the rhomboids in ${\mathbb {R}}^{d+1}$ by mapping every combinatorial vertex *Q* belonging to some rhomboid to $y_Q = \sum _{q \in Q} y_q$, in which $y_\emptyset = 0$, by convention. The $(d+1)$-st coordinate of $y_Q$ is therefore $- \#{Q}$, and we call $\#{Q}$ the *depth* of the vertex. The geometric realization of a rhomboid $\varrho$ is the convex hull of the locations of its combinatorial vertices, which is a rhomboid. We refer to ${{A}}_{ in}(\varrho )$ as the *anchor vertex* of $\varrho$.

**Fig. 2:**
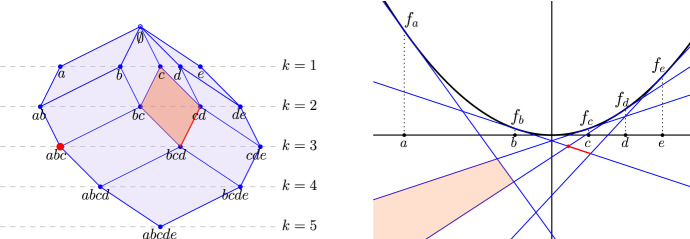
Left: the rhomboid tiling of five points in ${\mathbb {R}}^1$. The highlighted rhomboid defined by ${{A}}_{ in}= \{c\}$ and ${{A}}_{ on}= \{b,d\}$ is the convex hull of the points $y_c$, $y_{\{b,c\}}$, $y_{\{c,d\}}$, and $y_{\{b,c,d\}}$. The horizontal line at depth *k* intersects the tiling in the order-*k* Delaunay mosaic. Right: the dual hyperplane arrangement. Following the dotted lines connecting the points of *A* on the horizontal axis to the paraboloid, we find the corresponding tangent hyperplanes. The highlighted rhomboids of dimension $j = 0, 1, 2$ are dual to the highlighted cells of dimension $2-j$ in the arrangement

The *rhomboid tiling* of *A*, denoted $\mathrm{Rho}{({A})}$, is the collection of thus defined rhomboids. By assumption of general position, every face of a rhomboid is again defined by a sphere as described above and thus belongs to the rhomboid tiling. As proved in [11], any two rhomboids are either disjoint or intersect in a common face, which implies that the rhomboid tiling is a complex embedded in ${\mathbb {R}}^{d+1}$; see Fig. 2 for an example. The following properties have been observed in [11]:

### Proposition 1

(Rhomboid Tiling) Let $A \subseteq {\mathbb {R}}^d$ be locally finite and in general position.

1. $\mathrm{Rho}{({A})}$ is dual to an arrangement of hyperplanes in ${\mathbb {R}}^{d+1}$;
2. the slice of $\mathrm{Rho}{({A})}$ at depth *k* is the order-*k* Delaunay mosaic of *A*, scaled by a factor *k*.

**Fig. 3:**
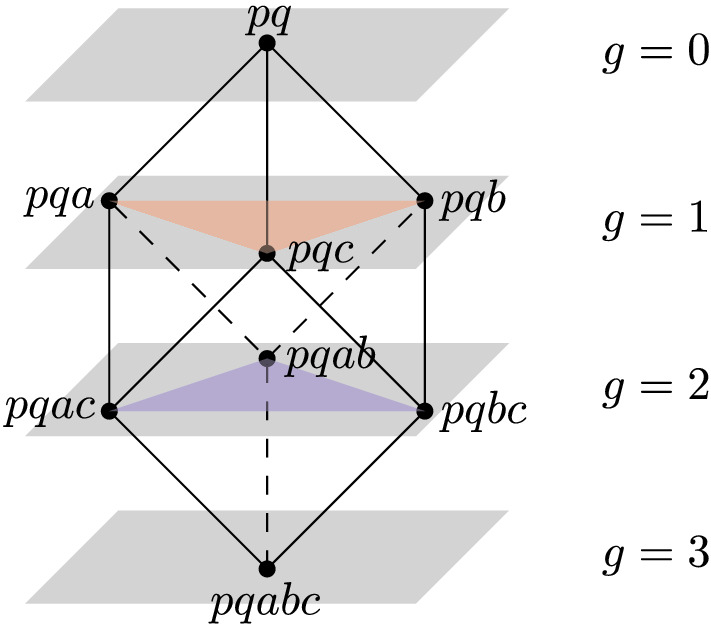
Slices of a $(d+1)$-dimensional rhomboid for $d=2$ defined by ${{A}}_{ in}(\varrho ) = \{p, q\}$ and ${{A}}_{ on}(\varrho ) = \{a,b,c\}$. The generation *g* of a slice of the rhomboid is the depth of the slicing plane relative to the anchor vertex of the rhomboid. For example, the slice in red is a *d*-dimensional cell of generation 1 (“first-generation slice” or “first-generation *d*-cell”) while the slice in blue is of generation *d*

**Fig. 4:**
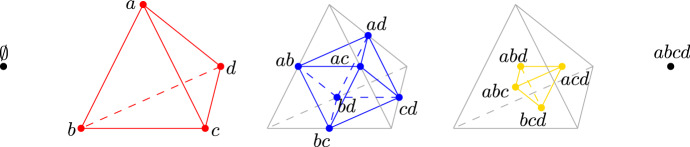
Slices of a 4-dimensional rhomboid defined by ${{A}}_{ in}(\varrho ) = \emptyset$ and ${{A}}_{ on}(\varrho ) = \{a,b,c,d\}$. The non-trivial slices are a tetrahedron at generation $g=1$, an octahedron at generation $g=2$, and another tetrahedron at generation $g=3$

The hyperplane arrangement will be introduced in Sect. 3.2. We elaborate on the second property: that each cell of the order-*k* Delaunay mosaic is a slice of some rhomboid. Combinatorially, each rhomboid is a cube and, again combinatorially, each cell of $\mathrm{Del}_{k}{({A})}$ is a slice orthogonal to the cube diagonal that passes through a non-empty set of the vertices. For the $(d+1)$-cube, there are $d+2$ such slices, which we index from top to bottom by the *generation* $0 \le g \le d+1$, see Fig. 3. The *g*-th slice passes through $$\left( {\begin{array}{c}d+1\\ g\end{array}}\right) $$ vertices, so we have a vertex at generations $g = 0,d+1$, a *d*-simplex at generations $g = 1,d$, and some other *d*-dimensional polytope at generations $2 \le g \le d-1$. In $d+1 = 3$ dimensions, we have a vertex, a triangle, another triangle, and another vertex, see Fig. 3; but already in $d+1 = 4$ dimensions, the middle slice is not a simplex; see Fig. 4. We remark that in addition to the order-*k* Delaunay mosaic, also the degree-*k* Delaunay mosaic, which is the dual of the degree-*k* Voronoi tessellation [12], can be obtained as a slice of $\mathrm{Rho}{({A})}$ at depth $k - \frac{1}{2}$.

## Combinatorial Properties

As proved in [4], the order-*k* Delaunay mosaic is the projection of the boundary complex of a convex polyhedron in ${\mathbb {R}}^{d+1}$. To explain this construction, we define the *lift* of $a \in {\mathbb {R}}^d$ as the point $\mathrm{lift}{({a})} = (a, \Vert {a}\Vert ^2) \in {\mathbb {R}}^{d+1}$. For each *k*-tuple $Q \subseteq A$, we take the barycenter of their lifts, $\frac{1}{k}\sum _{q\in Q} \mathrm{lift}{({q})}$, and obtain the order-*k* Delaunay mosaic as the vertical projection of the lower faces of the convex hull of these barycenters. Equivalently, we can interpret each barycenter of lifts as a weighted point in ${\mathbb {R}}^d$ and get the order-*k* Delaunay mosaic as the weighted order-1 Delaunay mosaic of the weighted points. By itself, this approach does not scale well with *k* since there are $$\left( {\begin{array}{c}\#{A}\\ k\end{array}}\right) $$ such barycenters. Most barycenters, however, are irrelevant as they do not contribute to the lower faces of the convex hull. If we could, somehow, identify the relevant barycenters without wasting time on the irrelevant ones, this procedure would efficiently construct the cells of the order-*k* Delaunay mosaic by computing the weighted first-order Delaunay mosaic. We will see how this can be done in Sect. 3.2.

In $d \ge 3$ dimension, not all cells of $\mathrm{Del}_{k}{({A})}$ are simplicial, even if the points in *A* are in general position. The cells carry important information, which for some applications is essential and cannot be easily recovered from a triangulation. This poses an additional challenge because most algorithms for computing convex hulls or weighted first-order Delaunay mosaics return a triangulated version of the correct mosaic. As explained in the following section, we address this issue by predicting the cells from their corresponding rhomboids.

### Predicting Cells

**Fig. 5:**
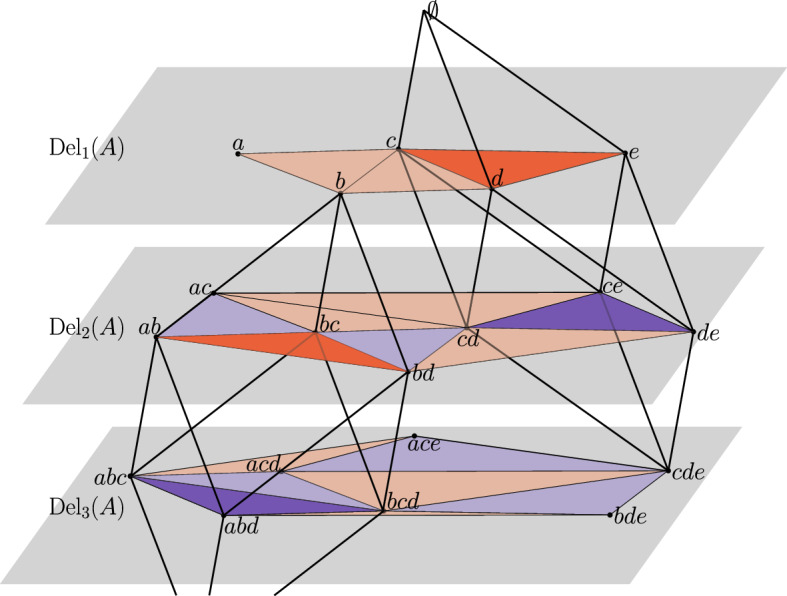
First-, second-, and third-order Delaunay mosaics of the set $A = \{a,b,c,d,e\}$ in ${\mathbb {R}}^2$ as slices of the 3-dimensional rhomboid tiling. For clarity, only two of the rhomboids are shown, with their first-generation slices in red and second-generation slices in dark blue. The rhomboids on the left and right are defined by ${{A}}_{ in}= \{b\}, {{A}}_{ on}= \{a,c,d\}$ and ${{A}}_{ in}= \emptyset , {{A}}_{ on}= \{c,d,e\}$, respectively (Color figure online)

Given a cell $\sigma$ in the order-*k* Delaunay mosaic, the following lemma identifies the rhomboid, $\varrho$, that $\sigma$ is a slice of; see Figs. 3 and 5 for an illustration. Write ${V}{({\sigma })}$ for the set of combinatorial vertices whose locations are the vertices of $\sigma$. Clearly, ${V}{({\sigma })} \subseteq {V}{({\varrho })}$.

#### Lemma 2

Let $\varrho \in \mathrm{Rho}{({A})}$ be a rhomboid and $\sigma \in \mathrm{Del}_{k}{({A})}$ a slice of $\varrho$. Then ${{A}}_{ in}(\varrho ) = \bigcap {V}{({\sigma })}$, ${{A}}_{ on}(\varrho ) = \bigcup {V}{({\sigma })} {\setminus } {{A}}_{ in}(\varrho )$, and the generation of $\sigma$ is $k - \#{{{A}}_{ in}(\varrho )}$.

#### Proof

Recall that ${V}{({\varrho })} = \{{{A}}_{ in}(\varrho ) \subseteq Q \subseteq {{A}}_{ in}(\varrho ) \cup {{A}}_{ on}(\varrho ) \}$, in which ${{A}}_{ in}(\varrho )$ and ${{A}}_{ on}(\varrho )$ are disjoint. Since the depth of a vertex is determined by its cardinality, and the vertices of a slice are by definition all at the same depth, the vertices of the generation-*g* slice all satisfy $\#{Q} - \#{{{A}}_{ in}(\varrho )} = g$. The intersection of all *g*-subsets of ${{A}}_{ on}(\varrho )$ is of course empty, which implies that the intersection of the combinatorial vertices of the slice is ${{A}}_{ in}(\varrho )$. Furthermore, $\bigcup {V}{({\sigma })} = \bigcup {V}{({\varrho })}$ for every slice $\sigma$ of $\varrho$ with generation $g \ge 1$. The union of all *g*-subsets of ${{A}}_{ on}(\varrho )$ is ${{A}}_{ on}(\varrho )$ itself, and thus ${{A}}_{ on}(\varrho ) = \bigcup {V}{({\sigma })} {\setminus } {{A}}_{ in}(\varrho )$. Finally, the generation of $\sigma$ is the difference in depth of the anchor vertex, ${{A}}_{ in}(\varrho )$, and the slice defining $\sigma$. The depth of $\sigma$ is *k* and the depth of ${{A}}_{ in}(\varrho )$ is its cardinality, which completes the proof. $\square$

If all of our order-*k* Delaunay cells are triangulated—e.g. due to being the output of a weighted first-order Delaunay algorithm—we cannot directly apply Lemma 2. Indeed, if $\tau$ is a simplex that is part of a triangulation of a slice $\sigma$ of a rhomboid $\varrho$, then $\bigcap {V}{({\tau })}$ and $\bigcup {V}{({\tau })}$ do not necessarily equal ${{A}}_{ in}(\varrho )$ and ${{A}}_{ in}(\varrho ) \cup {{A}}_{ on}(\varrho )$. We can, however, still identify whether $\tau$ is a first-generation slice of $\varrho$ and thus in fact is equal to $\sigma$. Using Lemma 2, we can then obtain $\varrho$.

#### Lemma 3

A *d*-simplex, $\tau$, in a triangulation of $\mathrm{Del}_{k}{({A})}$ is a first-generation *d*-cell of $\mathrm{Del}_{k}{({A})}$ if and only if the intersection of its combinatorial vertices is of size $k-1$.

#### Proof

Let $\sigma$ be the *d*-cell in $\mathrm{Del}_{k}{({A})}$ that contains $\tau$ in its triangulation, and assume $\sigma$ is a generation-*g* slice of $\varrho$. From Lemma 2, we know that ${{A}}_{ in}(\varrho ) \subseteq v$ for all $v \in {V}{({\sigma })}$, and $\#{{{A}}_{ in}(\sigma )} = k-g$. The remaining *g* points in every *v* are from ${{A}}_{ on}(\varrho )$. We have ${V}{({\tau })} \subseteq {V}{({\sigma })}$ with $\#{{V}{({\tau })}} = d+1$. So for $\tau$ to consist of vertices whose common intersection is of size $k-1$, there need to be $d+1$ distinct *g*-subsets of ${{A}}_{ on}(\varrho )$ that all have $g-1$ points in common. However, as $\#{{{A}}_{ on}(\varrho )} = d+1$, this is not possible unless $g=1$. $\square$

### Identifying Vertices

Given a triangulation of the order-*k* Delaunay mosaic, we just saw how to identify its first-generation *d*-cells. From these, we can obtain the corresponding rhomboids that these cells are slices of via Lemma 2, and their higher-generation slices by definition, also see Fig. 3. We shall now prove that if we have triangulations of the order-*j* Delaunay mosaics, for all $j < k$, we can assemble the complete vertex set of the order-*k* Delaunay mosaic by taking slices at depth *k* obtained from first-generation cells at lower depths. The key observation here is that for $k \ge 2$, there are no vertices in $\mathrm{Del}_{k}{({A})}$ whose incident *d*-cells are all of generation 1, also see Fig. 5. We note that this only holds in the unweighted setting.

To prepare the proof of this result, we recall the definition of the hyperplane arrangement postulated by Proposition 1. For each point $a \in A$, write $f_a :{\mathbb {R}}^d \rightarrow {\mathbb {R}}$ for the affine map defined by $f_a (x) = 2 {\langle x , a \rangle } - \Vert {a}\Vert ^2 = \Vert {x}\Vert ^2 - \Vert {x}-{a}\Vert ^2$. The graph of $f_a$ is a hyperplane in ${\mathbb {R}}^{d+1}$ that is tangent to the paraboloid ${\mathcal P}$ of points $(x,z) \in {\mathbb {R}}^d \times {\mathbb {R}}$ that satisfy $z = \Vert {x}\Vert ^2$. The collection of such hyperplanes decomposes ${\mathbb {R}}^{d+1}$ into convex cells, which we call the *hyperplane arrangement* of *A*, denoted $\mathrm{Arr}{({A})}$; see Fig. 2. The *cells* in the arrangement are intersections of hyperplanes and closed half-spaces. More formally, for each cell there is an ordered three-partition $A = {{A}}_{ in}\sqcup {{A}}_{ on}\sqcup {{A}}_{ out}$ such that the cell consists of all points $(x, z) \in {\mathbb {R}}^d \times {\mathbb {R}}$ that satisfy $z \le f_a (x)$, if $a \in {{A}}_{ in}$; $z = f_a (x)$, if $a \in {{A}}_{ on}$; and $z \ge f_a (x)$, if $a \in {{A}}_{ out}$. This three-partition is the key to establishing the bijection between the cells of $\mathrm{Arr}{({A})}$ and the rhomboids of $\mathrm{Rho}{({A})}$ that proves the duality claimed in Proposition 1, which is also illustrated in Fig. 2. We call top-dimensional cells of $\mathrm{Arr}{({A})}$ *chambers*; they satisfy ${{A}}_{ on}= \emptyset$. The depth of a chamber is $\#{{{A}}_{ in}}$ or, equivalently, the number of hyperplanes that are above this chamber; it equals the depth of the dual vertex in $\mathrm{Rho}{({A})}$. To see the aforementioned relationship between the arrangement and the higher-order Voronoi tessellations, we observe that the chamber in $\mathrm{Arr}{({A})}$ with ${{A}}_{ in}= Q$ vertically projects to $\mathrm{dom}{({Q})}$. We can therefore construct $\mathrm{Vor}_{k}{({A})}$ by computing and projecting all chambers whose ordered three-partitions satisfy $\#{{{A}}_{ in}} = k$; see [9, Chapter 13] or [12].

We call a chamber $\gamma$ a *bowl* if only one of its facets bounds it from above or, equivalently, if there is only one chamber at the next lower depth that shares a facet with $\gamma$. We call the hyperplane that contains this facet the *lid* of the bowl.

#### Lemma 4

A hyperplane that is a lid of a bowl at depth 1 is not a lid of any other chambers.

#### Proof

Let $\gamma$ be a bowl at arbitrary depth, and let *P* be its lid. Every other hyperplane that contains a facet of $\gamma$ bounds $\gamma$ from below. The top facet of $\gamma$ is the only part of *P* that is above all of these hyperplanes; that is: all other parts of *P* are below at least one of the other hyperplanes. This implies that every other bowl with lid *P* has at least one other hyperplane above it, and is thus of depth at least 2.

Now assume $\gamma$ is at depth 1. If there were another bowl $\gamma '$ with lid *P*, then the above argument would yield that all other bowls are at depth at least 2, contradicting our assumption on $\gamma$. Thus $\gamma$ has to be the unique bowl with lid *P*. $\square$

With this lemma, we are ready to state and prove the main combinatorial insight that motivates our algorithm. In a nutshell, it says that the first-generation cells form clusters without interior vertices. In ${\mathbb {R}}^2$, this is equivalent to saying that these clusters have outer-planar 1-skeletons.

#### Theorem 5

Let $A \subseteq {\mathbb {R}}^d$ be locally finite and $k \ge 2$. Then every vertex in $\mathrm{Del}_{k}{({A})}$ is vertex of some *d*-cell of generation $g \ge 2$.

#### Proof

In the unweighted setting, each hyperplane is tangent to the paraboloid ${\mathcal P}$ and contains a facet of the unique depth-0 chamber. Thus, each hyperplane is the lid to a chamber at depth 1. As this is true for every hyperplane, all chambers of depth 2 or higher have no lids by Lemma 4. This means that any chamber of depth at least 2 has at least two upper facets. Because the upper boundary is connected, there are two upper facets that meet in a $(d-1)$-face, the dual rhomboid of this face has dimension 2, and its bottom vertex is dual to the chamber. Thus we can obtain this vertex, *v*, knowing the other three vertices of the 2-rhomboid.

Any 2-rhomboid is a face of some $(d+1)$-dimensional rhomboid, $\varrho$, which thus contains *v* at generation at least 2, i.e. *v* has depth at least $\#{{{A}}_{ in}(\varrho )} + 2$. Knowing ${{A}}_{ in}(\varrho )$ and ${{A}}_{ on}(\varrho )$, we obtain this vertex via Eq. (1).

## Algorithm

We outline our algorithm in this section; its correctness follows from the results of the previous sections. Figure 5 visualizes the process, and Algorithm 1 gives a more formal write-up of the outline below.

We compute the Delaunay mosaics one by one in sequence of increasing order. We start by computing $\mathrm{Del}_{1}{({A})}$ using any existing algorithm for Delaunay triangulations. In this case, all *d*-cells are first generation *d*-cells. Using the definition of rhomboid slices as illustrated in Fig. 3, from these first generation *d*-cells we obtain second-generation slices which are *d*-cells of $\mathrm{Del}_{2}{({A})}$, third-generation slices which are *d*-cells of $\mathrm{Del}_{3}{({A})}$, and so on up to *d*th-generation slices. More generally, whenever we know the first-generation *d*-cells of $\mathrm{Del}_{j}{({A})}$, we compute the *g*th-generation *d*-cells for $g\le d$ from these, which are part of $\mathrm{Del}_{j+(g-1)}{({A})}$. By the time we need to compute $\mathrm{Del}_{k}{({A})}$ for some *k*, we will have already computed its *g*th-generation *d*-cells for $g\ge 2$, and only the first-generation *d*-cells are missing. By Theorem 5, from knowing the *g*th-generation *d*-cells for $g\ge 2$ we also know the complete vertex set of $\mathrm{Del}_{k}{({A})}$. From the vertex set of $\mathrm{Del}_{k}{({A})}$, we compute its (triangulated) *d*-cells (of any generation) using an off-the-shelf algorithm for weighted Delaunay triangulations, such as the Bowyer–Watson algorithm [5, 24] which underlies the CGAL implementation we use. We use Lemma 3 to identify the first-generation *d*-cells (which are simplices and thus not triangulated any further), while discarding all other cells. Together with the *g*th-generation *d*-cells for $g\ge 2$ which we obtained before, these give us the complete order-*k* Delaunay mosaic.

A dimension-agnostic python implementation of this algorithm and a 2- and 3-dimensional C++ implementation using CGAL [23] are available at [20, 21]. If we store all first-generation cells with their anchor vertices, we can use this algorithm to implicitly construct the rhomboid tiling, as done in [21].
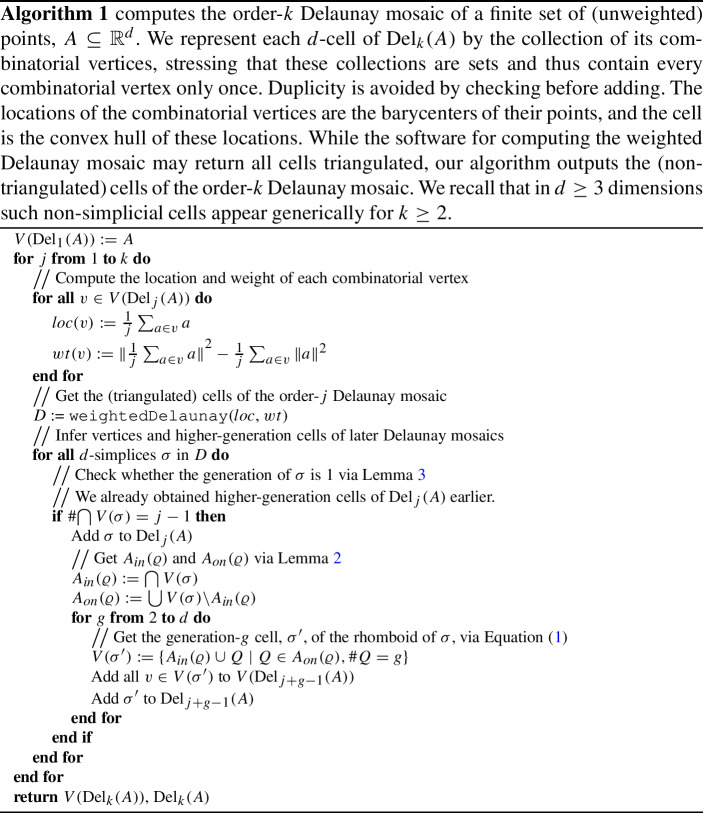


To get a handle on the runtime of the algorithm, we consider the two steps used to compute the order-*k* Delaunay mosaic after finishing the construction of the first $k-1$ mosaics. The first step is geometric and invokes the black-box algorithm to construct the weighted Delaunay mosaic from which we get vertices and cells of (unweighted) higher-order Delaunay mosaics. The runtime of this step depends on the runtime of the black box algorithm, which in many cases is output-dependent. The second step is combinatorial and determines, for each output simplex from the first step, whether it is first generation, in which case it is a genuine cell of the mosaic. Identifying whether an order-*k* cell is of first generation comes down to computing the intersection of $d+1$ sets of size *k* by Lemma 3, which can be done in expected *O*(*dk*). For those cells, obtaining their higher-generation cells takes time $O(2^d(k+d))$, as these higher generation cells have $O(2^d)$ vertices in total (they are a subset of the vertices of a $(d+1)$-dimensional rhomboid), and each vertex is a set of at most $k+d$ points. So assuming constant dimension, *d*, processing of each *d*-cell takes time *O*(*k*). Thus, for a given *k*, the combinatorial step takes time $O(k C_k)$, in which $C_k$ is the number of *d*-cells of $\mathrm{Del}_{k}{({A})}$. With each vertex being represented as a *k*-tuple of points, this is linear in the output size, assuming we store each cell naively as a set of its vertices. If the runtime of each black-box invocation were linear in the output size, the total runtime for producing the first *k* higher-order Delaunay mosaics would thus be linear in the output size as well.

In practice, it is more efficient to store a cell as a set of pointers or indices to its vertices, only requiring space $O(kV_k + C_k)$, with $V_k$ denoting the number of vertices of $\mathrm{Del}_{k}{({A})}$. Using this representation, the combinatorial step is not linear in the output size unless the number of cells of $\mathrm{Del}_{k}{({A})}$ is linear in the number of vertices.

## Experimental Results

In 2 dimensions, the number of cells in the (order-1) Delaunay mosaic is always linear in the number of input points, while in $d \ge 3$ dimensions, the size of the mosaic depends on the input set itself—and not just its cardinality—and ranges from $\Omega (n)$ to $O(n^{\lceil d/2 \rceil })$ [17]. The asymptotic worst case is realized by points located on the moment curve, $(t, t^2, \ldots , t^d)$ with $t \in {\mathbb {R}}$, while e.g. uniformly sampled points within a sphere have expected linear size [8], as do uniformly sampled points on a convex polytope in ${\mathbb {R}}^3$ [13]. Under appropriate sampling conditions for points on a smooth surface, the size of the mosaic is $O(n \log n)$ [3].

### Size in 3 dimensions

**Fig. 6:**
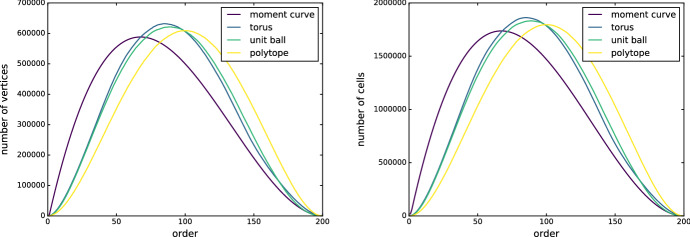
Number of vertices (left) and of 3-dimensional cells (right) in the order-*k* Delaunay mosaics of four sets with $n = 200$ points in ${\mathbb {R}}^3$ each

To shed light on the size range of order-*k* Delaunay mosaics, we compute them for a few 3-dimensional point sets relevant to these bounds. Note that for order-*k* Delaunay mosaics the number of vertices varies as well. Figure 6 shows the numbers of vertices and 3-dimensional cells for all higher-order Delaunay mosaics of four sets of size $n=200$ each: points on the moment curve, points sampled on the torus (with major radius 1 and minor radius 0.5 obtained by uniformly sampling the angles of its parametrization), points uniformly sampled inside the unit ball, and a point set in convex position forming a polytope (obtained by uniformly sampling points inside a ball and randomly choosing 200 vertices of the convex hull). The plots of vertex numbers and cell numbers generally resemble each other, with roughly three times as many cells as vertices. Other than in Fig. 6, we therefore omit the information about the vertices and show only the plots for the cells. The moment curve and polytope sets are both in convex position. Nevertheless, the size of the mosaic for the moment curve grows large faster for small *k*, and reaches its peak at $k \approx n/3$, while for the polytope the peak is at $k \approx n/2$. Notice how a faster rise also goes along with an earlier decay. This is a consequence of the fact that the total size of all order-*k* Delaunay mosaics together—or, equivalently of the rhomboid tiling—only depends on the input size, *n*, and not on the relative position of the input points.

### Size increase for small order

**Fig. 7:**
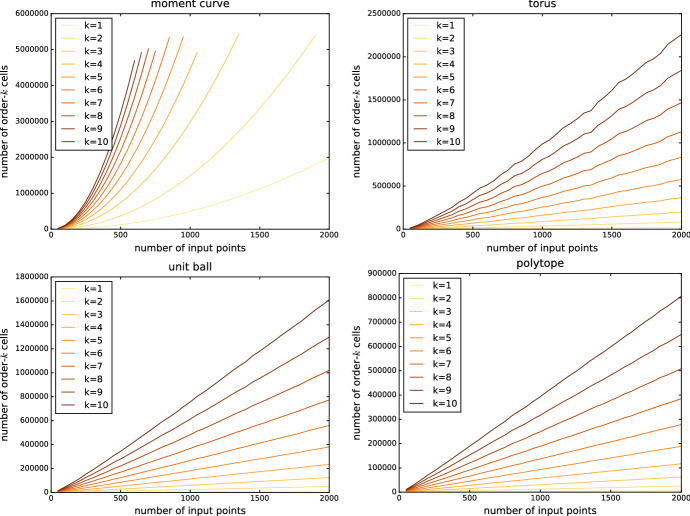
Number of cells in the order-*k* Delaunay mosaics for small *k* in relation to the input size, for various 3-dimensional point sets

Looking more closely at the growth for small *k* relative to the input size, we observe that the polytope and unit ball exhibit linear growth while the size of the mosaic seems to grow quadratically for the moment curve, see Fig. 7.

This is consistent with the bounds on first-order Delaunay mosaics mentioned earlier. For the torus, the size seems to grow slightly superlinearly, which is again consistent with the $O(n \log n)$ bound for smooth surfaces mentioned above.

### Variance

**Fig. 8:**
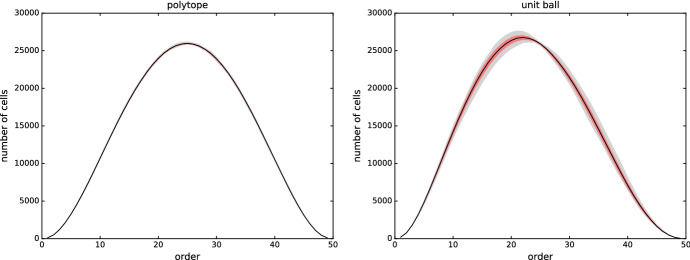
Variance of the number of 3-dimensional cells in the order-*k* Delaunay mosaics of randomly sampled points in convex position (left) and in a unit ball (right). The statistics of each plot are obtained from 30 sets of 50 points each. In black: the mean; in red: the range of one standard deviation around the mean; in grey: the range between the minimum and maximum (Color figure online)

To probe whether the above figures are representative, we investigate the variance in number of cells for the polytope and the unit ball. As shown in Fig. 8, the variance is particularly small for the polytope, and it is considerably larger of the unit ball. Curiously, the variance dips at $k = n/2$.

### Generations

**Fig. 9:**
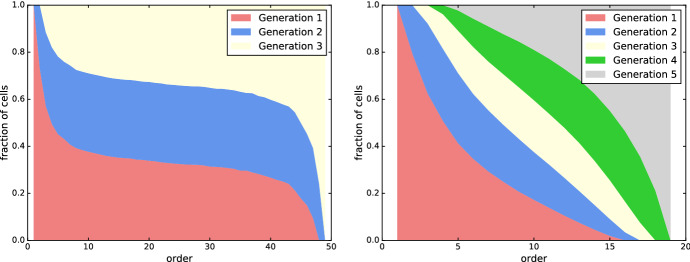
Fraction of cells of each generation in the order-*k* Delaunay mosaic, for 50 random points in the unit 3-ball (left) and 20 random points in the unit 5-ball (right)

We also investigate the distribution of cells of different generations. All point sets exhibit a pattern similar to that in Fig. 9, with the fraction of first-generation cells decreasing and the fraction of *d*-th-generation cells increasing as the order grows. The change is most prominent for small and large *k*, while the fractions remain almost constant in the range $k \approx n/2$, provided *n* is significantly larger than the dimension *d*.

### Curse of dimensionality

**Fig. 10:**
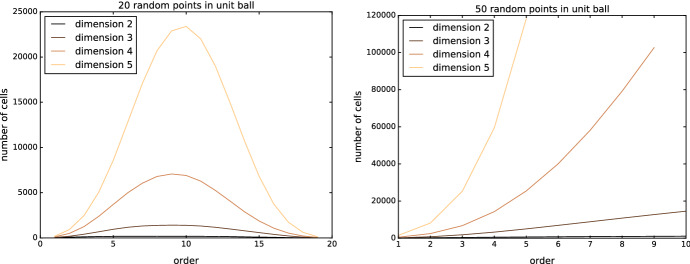
Number of *d*-cells in the order-*k* Delaunay mosaic for 20 points (left) and 50 points (right) randomly sampled in the unit ball for different dimensions *d*

Like many geometric structures, order-*k* Delaunay mosaics are subject to the dimensionality curse. Figure 10 shows how the size of order-*k* Delaunay mosaics behaves for point sets in different dimensions.

### Vertex degrees

**Fig. 11:**
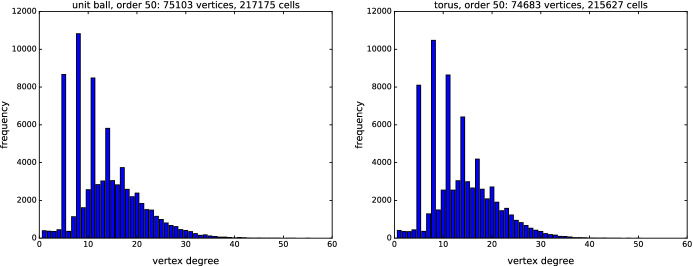
Vertex degree distribution in the order-50 Delaunay mosaic for 100 points sampled in the unit ball (left) and on the torus (right) in dimension 3

Order-*k* Delaunay mosaics in ${\mathbb {R}}^3$ exhibit an interesting distribution of vertex degrees for random point sets; see Fig. 11. The distribution looks like the sum of two distributions—with the second one only covering values 2 modulo 3—and is exhibited for all *k* except very small and very large ones. We do not know the reason for vertices being frequently incident to $5, 8, 11, \dots$ *d*-cells, but suspect these numbers correspond to geometric configurations of cells of different generations, such as three octahedra sharing a common vertex with two tetrahedra.

### Clusters

**Fig. 12:**
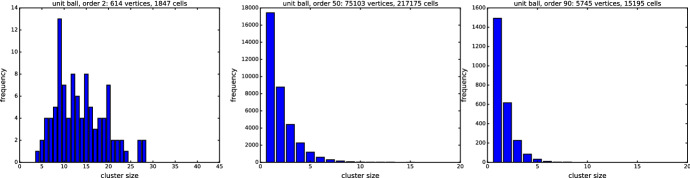
From left to right: distribution of cluster sizes in Delaunay mosaics of order 2, 50, and 90 for 100 random points in the unit ball

First-generation cells of any order-*k* Delaunay mosaic come in clusters connected by shared facets. We investigate the distribution of their sizes, leaving the discussion of their potential algorithmic significance for later. Figure 12 shows cluster size distributions in ${\mathbb {R}}^3$ for different orders. For very small *k*, the distribution depends on how the points are sampled, while for all other *k*, the cluster sizes seem to follow an exponential distribution. The decay rate increases with *k* and seems to be linked to the fraction of first-generation cells. It culminates in all clusters being singletons for $k = n-3$. For $k > n-3$, there are no more first-generation cells.

## Order-*k* Alpha Shapes

Beyond order-*k* Delaunay mosaics, our algorithm can be extended to compute *order-k alpha shapes*, as introduced in [15]. To this end, the rhomboid tiling is endowed with a radius function on its rhomboids [11]. It is inherited by the Delaunay mosaics, which are slices of the rhomboid tiling, and their sublevel sets with respect to this radius function are complexes that geometrically realize the order-*k* $\alpha$-shapes. In this section, we recall the definition of the radius function from [11], and present an efficient way of computing it.

To get started, we note that the radius function needs a representation for every rhomboid in the tiling, but the algorithm in Sect. 4 computes only the top-dimensional rhomboids. This is easily remedied by noticing that the dimension of a rhomboid is $k = \#{{{A}}_{ on}{({\varrho })}}$ and its $3^k$ faces correspond to the different ways of partitioning ${{A}}_{ on}{({\varrho })}$ into three sets. For the remainder of the discussion, assume that we have a representation for the rhomboids of all dimensions $0 \le j \le d+1$ in $\mathrm{Rho}{({{A}})}$. Each *j*-dimensional rhomboid, $\varrho \in \mathrm{Rho}{({{A}})}$, corresponds to a $(d+1-j)$-dimensional cell in the dual arrangement, $\varrho ^* \in \mathrm{Arr}{({{A}})}$. We introduce ${\mathcal P}_{t} ({x}) :{\mathbb {R}}^d \rightarrow {\mathbb {R}}$ defined by mapping $x \in {\mathbb {R}}^d$ to ${\mathcal P}_{t} ({x}) = \tfrac{1}{2} (\Vert {{x}}\Vert ^2 - t)$. With slight abuse of notation, we write ${\mathcal P}_{t}$ for the graph of this function. This graph is the paraboloid ${\mathcal P}_0$ dropped down vertically by a distance $\tfrac{t}{2}$. We define the *squared radius function* $\mathcal{R}^2 :\mathrm{Rho}{({{A}})} \rightarrow {\mathbb {R}}$, which maps a rhomboid to the minimum *t* such that ${\mathcal P}_{t}$ has a non-empty intersection with $\varrho ^*$. We call a sphere *constrained* by $\varrho$ if it encloses ${{A}}_{ in}{({\varrho })}$, passes through all points of ${{A}}_{ on}{({\varrho })}$, and has no other points of *A* inside. Letting ${S}_{ min}{({\varrho })}$ be the smallest such sphere, we get an alternative interpretation of the radius function:

### Lemma 6

$\mathcal{R}^2(\varrho )$ equals the squared radius of ${S}_{ min}{({\varrho })}$.

### Proof

The proof of Theorem 1 of [11] establishes a map from points in the $\mathrm{Arr}{({{A}})}$ to spheres: a point ${y}= ({x}, {z}) \in {\mathbb {R}}^d \times {\mathbb {R}}$ below the paraboloid ${\mathcal P}$ is mapped to the sphere, *S*, with center ${x}$ and squared radius $\Vert {{x}}\Vert ^2 - 2{z}$. Importantly, if $\varrho ^*$ is the cell in the dual arrangement whose interior contains ${y}$, then *S* is constrained by $\varrho$, which is the rhomboid dual to $\varrho ^*$.

Now let $t = \mathcal{R}^2(\varrho )$, and let $r^2$ be the squared radius of ${S}_{ min}{({\varrho })}$. By definition, *t* is the smallest value for which ${\mathcal P}_{t}$ contains a point ${y}\in \varrho ^*$. The aforementioned map maps ${y}$ to a sphere constrained by $\varrho$, thus $r^2 \le t$. When reversing this map, ${S}_{ min}{({\varrho })}$ is mapped to a point of $\varrho ^*$. As *t* was the smallest value for which ${\mathcal P}_{t}$ touches $\varrho ^*$, we have $t \le r^2$. Thus the squared radius of ${S}_{ min}{({\varrho })}$ equals $\mathcal{R}^2(\varrho )$. $\square$

To compute this radius function, we first get the smallest sphere constrained by a rhomboid. While Welzl’s algorithm [25] for smallest enclosing sphere can be adapted to this task, it takes *O*(*n*) with $n = \#{{A}}$ for each such sphere computation. To improve on this bound, we recall that Lemma 3 of [11] establishes that rhomboids of the same radius value come in intervals $[\varrho _{min}, \varrho _{max}] := \{\varrho \in \mathrm{Rho}{({{A}})} \mid \varrho _{min} \subseteq \varrho \subseteq \varrho _{max}\}$ whose lower bound, $\varrho _{min}$, is a vertex. To identify the vertex ${v}$ that a rhomboid $\varrho$ forms an interval with, we need to identify its vertex with the same radius value. By Lemma 6 this means the radii of ${S}_{ min}{({\varrho })}$ and ${S}_{ min}{({{v}})}$ have to be the same, and it is not difficult to see that the spheres ${S}_{ min}{({\varrho })}$ and ${S}_{ min}{({{v}})}$ are in fact the same. As ${{A}}_{ on}{({{v}})} = \emptyset$ for any vertex ${v}$, the sphere achieving the radius value of ${v}$ is defined solely by inclusions and exclusion constraints. Therefore all constraints of $\varrho$ that require points of ${{A}}_{ on}{({\varrho })}$ to be on the sphere need to be converted to inclusion and exclusion constraints without affecting the resulting sphere. We know that such constraints exist because the lower bound of the interval is a vertex. This observation gives rise to the following lemma.

### Lemma 7

Let $\varrho$ be a rhomboid that is an upper bound of an interval. Let ${A}_I \subseteq {{A}}_{ on}{({\varrho })}$ such that the smallest enclosing sphere *S* of ${A}_I$ that excludes ${{A}}_{ on}{({\varrho })} {\setminus } {A}_I$ is the same as the circumsphere of ${{A}}_{ on}{({\varrho })}$. Then $\varrho$ forms an interval with the vertex ${v}= {{A}}_{ in}{({\varrho })} \cup {A}_I$.

### Proof

As $\varrho$ is an upper bound of an interval, its sphere, ${S}_{ min}{({\varrho })}$, is only supported by ${{A}}_{ on}{({\varrho })}$. Indeed, if there were another point ${a}\in {{A}}_{ in}{({\varrho })}$—or ${a}\in {{A}}_{ out}{({\varrho })}$—on the surface of this sphere, then the rhomboid $\varrho$ with ${{A}}_{ on}{({\varrho })} = {{A}}_{ on}{({\varrho })} \cup \{{a}\}$ and ${{A}}_{ in}{({\varrho })} = {{A}}_{ in}{({\varrho })} {\setminus } \{{a}\}$—or ${{A}}_{ out}{({\varrho })} = {{A}}_{ out}{({\varrho })} {\setminus } \{{a}\}$—would be a higher-dimensional rhomboid with the same sphere ${S}_{ min}{({\varrho })} = {S}_{ min}{({\varrho })}$, contradicting that $\varrho$ be an upper bound of an interval.

As ${S}_{ min}{({\varrho })}$ is only supported by ${{A}}_{ on}{({\varrho })}$, this means that ${S}_{ min}{({\varrho })}$ is the same as the circumsphere of ${{A}}_{ on}{({\varrho })}$, which by our assumption is the same as *S*. Now the inclusion and exclusion constraints of *S* are part of the constraint set for ${S}_{ min}{({{v}})}$, but because $S = {S}_{ min}{({\varrho })}$ it does in fact fulfill all the constraints of ${S}_{ min}{({{v}})}$. Thus ${S}_{ min}{({{v}})} = S = {S}_{ min}{({\varrho })}$, proving that they are in the same interval. $\square$

### Algorithm

Assume $\varrho$ is a *j*-rhomboid that is an upper bound of an interval. Let *S* be the circumsphere of ${{A}}_{ on}{({\varrho })}$. For each point ${a}\in {{A}}_{ on}{({\varrho })}$, we need to decide whether to impose an inclusion or exclusion constraint on it. Let $S_{a}$ be the circumsphere of ${{A}}_{ on}{({\varrho })} {\setminus } \{{a}\}$. If ${a}$ is outside of $S_{a}$, then imposing an exclusion constraint for ${a}$ would yield $S_{a}$ rather than *S*, thus we add ${a}$ to ${A}_I$ in order to impose an inclusion constraint for it. Similarly, if ${a}$ is inside of $S_{a}$, we have to impose an exclusion constraint for ${a}$ and thus do not add it to ${A}_I$.

While this is difficult for an individual rhomboid, it becomes straightforward if we compute all intervals in the rhomboid tiling. We know that all $(d+1)$-rhomboids are upper bounds of intervals. After marking all rhomboids that are contained in such intervals, we know that all remaining unmarked *d*-rhomboids are upper bounds of intervals. Thus by processing the rhomboids in decreasing dimension, all unmarked rhomboids we encounter are upper bounds.

## Discussion

This paper presents a simple algorithm for computing order-*k* Delaunay mosaics in Euclidean space of constant dimension. Implementations of the algorithm—in C++ for points in ${\mathbb {R}}^2$ and ${\mathbb {R}}^3$ and in python for points in ${\mathbb {R}}^d$—are provided [20, 21]. This software includes the application to the persistence of *k*-fold covers described in [11]. The remainder of this section discusses this application and possible extensions and optimizations of our algorithm.

### *k*-fold covers

The sublevel sets of the order-*k* Delaunay mosaics with respect to the radius function introduced in Sect. 6 are homotopy equivalent to *k*-fold covers of Euclidean balls. It follows that our algorithms facilitate the computation of persistence of these *k*-fold covers. Furthermore, the circumcenters of the spheres that are used in the computation of the radius function provide the geometric locations of the order-*k* Voronoi vertices and allow reconstructing the order-*k* Voronoi tessellation via duality.

### Weighted setting

Our algorithm generalizes to points with real weights, but not easily. The main challenge is the extraction of the vertices of the order-*k* mosaic from lower-order mosaics. This extraction relies on Theorem 5, which does not hold for weighted points. Indeed, a crucial assumption in this theorem is that every lifted hyperplane is incident to the depth-0 chamber of the arrangement, and this property is generally violated for weighted points. This is the same assumption used in the prior dimension-agnostic algorithms [1, 18, 19]. For sets of weighted points that satisfy this assumption, our algorithm and these prior algorithms still work. To overcome this limitation, we would need a way to detect all bowls in the arrangement, because they correspond to the vertices in the Delaunay mosaics our algorithm is not able to find. Identifying the bowls is an independent problem, and any solution to it can be combined with our algorithm. Once we know the bowls and add the corresponding vertices to the appropriate mosaics, our algorithm works as before.

### Clusters of cells

As mentioned in Sect. 5, first-generation cells in the order-*k* Delaunay mosaic are organized in clusters. To formally define them, consider the graph whose nodes are the cells and whose arcs are the shared facets (i.e. the 1-skeleton of the order-*k* Voronoi tessellation). A *cluster* is a connected component in the subgraph induced by the first-generation cells. It is not difficult to see that two such cells belong to a common cluster if and only if the corresponding rhomboids have the same anchor vertex. Let $\varrho$ be one of these rhomboids and recall that the anchor vertex is ${{A}}_{ in}(\varrho )$, which in this case is a collection of $k-1$ points of *A*. Each combinatorial vertex of any cell in the cluster contains these $k-1$ points, plus one additional point, which differentiates between these vertices. In other words, the cluster as a subcomplex of the order-1 Delaunay mosaic of these additional points.

With this insight, we could replace the weighted Delaunay mosaic of the entire vertex set by multiple instances of unweighted Delaunay mosaics, namely one per cluster. This alternative strategy avoids the need to compute averages of points at the cost of extra book-keeping to group the vertex set of $\mathrm{Del}_{k}{({A})}$ into clusters. We mention that in ${\mathbb {R}}^2$, the structure of each cluster satisfies the requirements that allow for the construction in time linear in the number of points [2].

### Exact arithmetic

The CGAL software library [23] supports exact arithmetic by distinguishing between *exact constructions* and *exact predicates*. The latter are geometric tests with a true or false answer, such as whether or not a given point lies on a given sphere. By itself, the CGAL algorithm for weighted Delaunay triangulations requires exact predicates but no exact constructions. Our algorithm, on the other hand, computes averages of collections of input points, which are the locations of the vertices of the mosaic. This is an exact construction and indeed the only one needed to run our algorithm with exact arithmetic. In practice, exact constructions are a significant overhead with noticeable impact on the runtime, which would be nice to avoid.
